# More Physicians and Dentists Should Participate in Clinical Trials Now!

**DOI:** 10.7759/cureus.113102

**Published:** 2026-07-21

**Authors:** Gerald L Klein, Marion Stamp-Cole, Freddy Byrth, Gail Brown, Patrick Loebs, Roger E Morgan, Kenny Carberry, Alexandre Vieira

**Affiliations:** 1 Medical Affairs, MedSurgPI, Pittsboro, USA; 2 Clinical Operations, MSC Clinical Consulting, Shelbyville, USA; 3 Clinical Operations, MedSurgPI, Pittsboro, USA; 4 Clinical Research Operations, MedSurgPI, Pittsboro, USA; 5 Oncology, The Jackson Brown Group, San Jose, USA; 6 Clinical Research/Drug Development, MedSurgPI, Pittsboro, USA; 7 Clinical Operations, Carberry Clinical, Franklin, USA; 8 School of Dental Medicine, East Carolina University, Greenville, USA

**Keywords:** clinical trials, clinical trials in all phases (i-iv), dental research, medical innovation, patient access, physician participation, practice growth, professional development, research in clinical trials

## Abstract

Clinical trials offer physicians and dentists meaningful opportunities to expand patient care, strengthen professional relevance, and contribute directly to scientific progress. Many patients, particularly those with chronic, progressive, rare, or treatment‑resistant conditions, exhaust standard therapies and may benefit from investigational options available only through research. By participating in clinical trials, clinicians might provide access to emerging therapies, diagnostics, devices, and care strategies while allowing patients to remain within their trusted community practices. Beyond patient benefit, clinical trial involvement may enhance professional identity by positioning clinicians as contributors to evidence generation rather than solely consumers of it. Participation may increase referrals, assist in elevating a practice’s reputation, and create opportunities for scholarly collaboration, including publications and conference presentations. When properly structured, research may also provide legitimate financial value through study budgets that offset investigator time, staff effort, and protocol‑required procedures. Engagement in clinical research potentially exposes clinicians to new scientific developments earlier, offering a dynamic form of continuing education that may help keep their practice intellectually stimulating. For many, research participation renews professional purpose and may help mitigate burnout by reconnecting daily work to discovery, teamwork, and broader contributions to patient care. As healthcare moves toward precision medicine, AI‑enabled discovery, and pragmatic real‑world trials, clinician participation across all practice settings will be increasingly necessary and essential.

## Editorial

Executive summary

Clinical trials may offer physicians and dentists several advantages: more options for patients, earlier access to emerging science, and a practical way to differentiate and grow a practice. When research is well supported and ethically managed, participation may help expand treatment pathways, strengthen clinical relevance, and create operational value for community and specialty practices alike.

Clinical trials are research studies involving human volunteers and patients that evaluate medications, vaccines, devices, procedures, or other interventions for safety and effectiveness [1]. They are central to medical advancement because they test new ways to prevent, detect, and treat disease [2]. For physicians and dentists, participating in clinical research may extend patient care by connecting everyday practice to new treatment options, scientific progress, and measurable practice development across a wide range of care settings.

Helping patients access more options

One of the strongest reasons physicians and dentists should participate in clinical trials is that trials may give patients more options for medical treatment, as well as potentially extending care for underserved populations. Standard-of-care therapies that are refractory or resistant may not work for every patient, and many patients face chronic, progressive, rare, or treatment‑resistant conditions. For these individuals, a clinical trial may offer access to an investigational therapy, device, diagnostic approach, or care strategy that is not yet widely available.

The National Cancer Institute notes that clinical trial participation may give patients access to a treatment under study, provide closer monitoring by a research team, and help researchers contribute knowledge that may benefit future patients [3]. This is especially meaningful in community-based practice, where patients often prefer to receive care close to home from physicians they already know and trust.

Physicians and dentists who participate in trials may become more than providers of standard care. They may become advocates for expanded access, scientific progress, and therapeutic possibility. For patients who feel they have exhausted available standard-of-care therapies, the impact can be deeply meaningful.

Financial and practice-building benefits

Clinical trials should always be conducted ethically and with patient welfare as the highest priority. However, when properly managed, research also has the potential to create legitimate financial value for a professional practice. Trial budgets may supplement investigator time, study coordinator activities, patient visits, regulatory documentation, data entry, screening procedures, and protocol-required assessments. At the same time, startup costs, compliance obligations, and staffing needs can be significant, especially for practices that are new to research.

Medicare may also cover routine costs of qualifying clinical trials, as well as reasonable and necessary services used to diagnose and treat complications arising from participation, subject to applicable Medicare rules. [4] This coverage framework helps support the integration of research into clinical care.

Beyond direct revenue, trials can help build a practice, although results vary by specialty, patient population, sponsor fit, and operational readiness. A research-active practice may attract patients seeking advanced treatment options, increase referrals from physicians who do not offer trials, strengthen relationships with hospitals or health systems, and develop a reputation as a site for innovative care. In a competitive healthcare environment, clinical research can help differentiate a practice, but it typically requires reliable staffing, regulatory discipline, and careful study selection to be sustainable.

Professional prestige and clinical leadership

Participation in clinical trials can strengthen the professional identity of physicians and dentists by positioning them as active contributors to advancing medical knowledge. Physicians and dentists involved in research are not simply applying evidence generated elsewhere; they are helping create that evidence. This may elevate their standing with patients, peers, hospitals, sponsors, and referring providers.

Research has found that providers participate in community oncology clinical trials for both altruistic and professional reasons, including increasing patient access to trials, fulfilling an obligation to patients, improving quality of care, and enhancing organizational or professional reputation [5]. These benefits are not limited to academic medical centers. Community physicians are also recognized as leaders in innovation and access, as patients may receive new therapies without having to travel to major medical centers.

Clinical trial participation may also create opportunities that extend well beyond the clinic, although these vary by specialty, experience, and level of research involvement. Some investigators are invited to co-author peer-reviewed publications, present at scientific conferences, or contribute to advisory activities and protocol development. For physicians and dentists who build sustained research experiences, these roles can strengthen professional credibility and expand their contribution to the evolution of care.

Staying on the cutting edge of science

Medicine and oral healthcare change quickly. New drugs, biologics, devices, biomaterials, diagnostics, digital workflows, and treatment pathways are constantly emerging. Physicians and dentists who participate in clinical trials are exposed to these developments earlier and more deeply than those who encounter them only after approval, publication, or broad adoption.

The National Institutes of Health (NIH) describes clinical trials as being “at the heart of all medical advances” and notes that they may study new drugs, new combinations of drugs, new surgical methods, medical devices, new uses of existing treatments, behavioral interventions, and ways to improve quality of life [2]. This breadth makes clinical research a powerful form of continuing education.

For some physicians and dentists, trials may provide intellectual stimulation grounded in day-to-day practice. This requires careful thinking about disease mechanisms, patient selection, endpoints, safety monitoring, adverse events, workflow integration, and evolving standards of care. It can make everyday practice more engaging while helping clinicians stay active learners throughout their careers. Key reasons for participation are summarized in Table 1.

**Table 1:** Key reasons physicians and dentists should participate in clinical trials Sources: [6-8]

Benefit	Why it matters	Practical impact for physicians and dentists
Financial opportunity	Clinical trials may create an additional revenue stream when properly budgeted and managed.	May help support investigator time, staff time, patient visits, regulatory work, and study-related procedures.
Professional prestige	Sometimes trial participation positions physicians as leaders in medical innovation.	Potential to enhance reputation with patients, peers, hospitals, sponsors, and referring providers.
More options for patients	Trials may give patients access to new therapies, devices, diagnostics, or care strategies before they are widely available.	May allow physicians and dentists to offer additional choices, especially when standard treatments are limited.
Cutting edge of science	Physicians and dentists involved in trials are sometimes able to see emerging therapies and scientific advances earlier.	Keeps clinicians current on new drugs, biomarkers, protocols, endpoints, and standards of care.
Practice growth	Research may sometimes differentiate a practice in a competitive market.	Can frequently attract new patients, increase referrals, and strengthen relationships with sponsors and health systems.
Renewed interest in medicine and dentistry	Clinical trials should add intellectual challenge and variety to routine clinical practice.	This can help physicians stay engaged, curious, and professionally energized.
Burnout prevention	Research may reconnect physicians with purpose, discovery, and patient impact.	Can add meaning to practice and may reduce feelings of routine, stagnation, or professional fatigue.

Renewing interest in medicine and dentistry

Many people enter medicine and dentistry because they are curious, service-oriented, and motivated by discovery. Over time, however, the realities of clinical practice, including administrative burdens, documentation demands, productivity pressures, and repetitive workflows, can make medicine feel less inspiring.

Participating in clinical trials might be a path that can help restore a sense of curiosity and mission. They introduce new questions, new protocols, new therapies, and new collaborations into daily practice. They allow physicians to participate in the future of medicine rather than simply respond to it after the fact.

This renewed engagement might be especially important for experienced physicians who want to keep growing professionally. Research may provide a new dimension of practice without requiring a physician to leave clinical care.

Helping prevent burnout

Physician and dentist burnout are major concerns in modern healthcare. The Agency for Healthcare Research and Quality (AHRQ) defines burnout as a long-term stress reaction marked by emotional exhaustion, depersonalization, and a reduced sense of personal accomplishment [9]. Clinical trials are not a cure for burnout, and poorly supported research can add stress. However, when trials are well organized and supported by appropriate research-oriented infrastructure, they may help address some of the drivers of professional dissatisfaction.

Participating in research may provide physicians and dentists a sense of contribution beyond the exam room. It connects daily work to larger scientific progress. It might also create a team-based environment involving investigators, coordinators, nurses, sponsors, monitors, and patients working toward a shared goal. For some clinicians, this sense of purpose may make medical practice feel more meaningful and less transactional.

The Association of Clinical Research Professionals (ACRP) has noted that clinical research may provide physicians and dentists with benefits such as more options for patients, additional revenue, and job satisfaction, while also acknowledging that practices need proper support to manage the operational burden of research [10]. A distinction needs to be made among the challenges faced by the solo practitioner, the multi‑provider practice owner, and the practitioner who is an employee of a larger institution, as each group encounters different operational and structural barriers. All the problems will be different; this still affects all practitioners whether they are solo, multi-provider practice, or employees of larger institutions. This point is important: clinical trials are most beneficial when physicians and dentists have the right systems, staffing, and partners in place.

Alignment with the nation's needs

Participating in clinical trials helps bridge the gap between basic laboratory science and real‑world patient care. This is important because it will accelerate the translation of research into practice, improve health outcomes, and promote the development of accessible, effective, and safe treatments. Population needs may be better addressed by translating research findings and delivering evidence‑based interventions across all healthcare settings. The future lies in precision medicine and dentistry, AI‑driven drug discovery, and real‑world pragmatic trials.

Conclusion

More physicians and dentists should participate in clinical trials because doing so may expand patient options, keep clinicians closer to emerging science, and help practices grow in credible, practical ways. When supported by the right infrastructure, research participation may benefit patients, strengthen clinical positioning, and add value to both community and specialty care settings.

At its best, clinical research has the potential to allow physicians and dentists to improve care today while contributing to better care tomorrow. For clinicians seeking to broaden treatment options, stay current, and build a more differentiated practice, trial participation remains a meaningful and practical path.
